# Coblopasvir and sofosbuvir for treatment of chronic hepatitis C virus infection in China: A single‐arm, open‐label, phase 3 trial

**DOI:** 10.1111/liv.14633

**Published:** 2020-10-13

**Authors:** Yanhang Gao, Fei Kong, Guangming Li, Cheng Li, Sujun Zheng, Jianmei Lin, Xiaofeng Wen, Jinghua Hu, Xiaozhong Wang, Xiaofeng Wu, Huichun Xing, Jidong Jia, Zhansheng Jia, Yujuan Guan, Chenghao Li, Guicheng Wu, Zhiliang Gao, Zhuangbo Mou, Qin Ning, Qing Mao, Yongfeng Yang, Jing Ning, Li Li, Hai Pan, Desheng Zhou, Yanhua Ding, Hong Qin, Junqi Niu

**Affiliations:** ^1^ Department of Hepatology the First Hospital of Jilin University Changchun China; ^2^ Cirrhosis Department Zhengzhou Sixth Municipal People’s Hospital Zhengzhou Henan China; ^3^ Difficult & Complicated Liver Diseases and Artificial Liver Center Beijing You An Hospital Capital Medical University Beijing China; ^4^ Department of Infectious Diseases Sichuan Provincial People’s Hospital Chengdu Sichuan China; ^5^ Department of Hepatology Liuzhou People’s Hospital Liuzhou China; ^6^ Liver Failure Treatment and Research Center the Fifth Medical Center of PLA General Hospital Beijing China; ^7^ Department of Hepatology Xinjiang Uygur Autonomous Region Traditional Chinese Medicine Hospital Urumqi Xinjiang China; ^8^ Department of Hepatology Shenyang Sixth People’s Hospital Shenyang, Liaoning China; ^9^ Department of Hepatology Division 3 Beijing Ditan Hospital Capital Medical University Beijing China; ^10^ Liver Research Center Beijing Youyi Hospital Affiliated to Capital Medical University Beijing China; ^11^ Department of Infectious Diseases the Second Affiliated Hospital of People’s Liberation Army Air Force Medical University Xi’an, Shaanxi China; ^12^ Department of Hepatology Guangzhou Eighth People’s Hospital Guangzhou China; ^13^ Department of Gastroenterology Yanbian University Affiliated Hospital Yanji Jilin China; ^14^ Department of Hepatology Chongqing University Three Gorges Hospital Chongqing Three Gorges Central Hospital Wanzhou, Chongqing China; ^15^ Department of Infectious Diseases the Third Affiliated Hospital of Dr Sun Yat‐Sen University Guangzhou Guangdong China; ^16^ Department of Hepatology Ji’nan Municipal Hospital of Infectious Diseases Ji’nan, Shandong China; ^17^ Department of Infectious Diseases Tongji Hospital Affiliated to Tongji Medical College Huazhong University of Science and Technology Wuhan Hubei China; ^18^ Institute of Infectious Diseases the First Affiliated Hospital of People’s Liberation Army Medical University Chongqing China; ^19^ Department of Hepatology Nanjing Second Municipal Hospital Nanjing China; ^20^ Research and Development Center Beijing Kawin Technology Share‐Holding Co., Ltd Beijing China; ^21^ The Department of Phase I Clinical Trial the First Hospital of Jilin University Changchun, Jilin China; ^22^ Present address: Clinical Development Hangzhou Sciwind Biosciences Co., Ltd Hangzhou Zhejiang China

**Keywords:** coblopasvir, pangenotypic regimen, safety, sofosbuvir, sustained virological response

## Abstract

**Background & Aim:**

An affordable, pangenotypic regimen remains as an unmet medical need for chronic hepatitis C patients in China. This single‐arm, open‐label, multicenter, phase 3 trial evaluated the efficacy and safety of coblopasvir, a pangenotypic non‐structural protein 5A (NS5A) inhibitor, combined with sofosbuvir for treating Chinese patients with chronic hepatitis C virus (HCV) infection.

**Methods:**

Treatment‐naïve and interferon‐experienced adult patients, including those with advanced fibrosis (F3) or compensated cirrhosis (F4), were treated with a universal, combinational regimen of coblopasvir 60 mg and sofosbuvir 400 mg, once daily, for 12 weeks. The primary efficacy endpoint was sustained virological response at post‐treatment week 12 (SVR12).

**Results:**

Overall, 371 patients (men, 51%; age, 47 ± 11 years; genotype 1a < 1%, 1b 48%, 2a 26%, 3a 6%, 3b 7% and 6 12%) were enrolled from 19 sites. Fifty‐one patients (14%) had F3, 39 patients (11%) had F4 and 39 patients (11%) were interferon experienced. The overall SVR12 was 97% (95% CI, [94%, 98%]) for the full analysis set and was equal to or above 90% for all predefined subsets. Ten patients (3%) experienced virological relapse and two patients did not complete follow‐up. No adverse events (AEs) occurred at a frequency ≥5%, and the most often reported AEs (≥1%) were neutropenia and fatigue. The majority of AEs were mild to moderate and transient without specific medical intervention.

**Conclusions:**

The universal, pangenotypic combo of coblopasvir plus sofosbuvir is an efficacious and safe treatment for Chinese patients monoinfected with HCV of genotype 1, 2, 3 and 6, including those with compensated cirrhosis.

**Lay summary:**

The regimen of coblopasvir and sofosbuvir is a safe and effective treatment for Chinese patients with genotype 1, 2, 3 and 6 HCV infection, including those with compensated cirrhosis. Therefore, this regimen would be a novel choice of treatment for this patient population.

## 1 INTRODUCTION

China has a high prevalence of hepatitis C virus (HCV) infection, with an estimated infected population of at least 10 million.^1^ HCV genotype distribution is also highly diverse across the nationwide geographical regions, with genotype 1b being the most dominant. Genotype 2 is more frequent in Northern China and genotypes 3 and 6 are more common in Southern China.^2^ Furthermore, genotype 3b, a subtype specific to China, differs from subtype 3a in virological response to direct‐acting antivirals (DAAs).^3^ Therefore, an accessible, potent, standard‐course, pangenotypic treatment regimen remains an unmet medical need in China from the perspectives of both clinical practice and public health, although two imported pangenotypic fixed‐dose combinations (velpatasvir‐sofosbuvir and glecaprevir‐pibrentasvir) have been conditionally approved by the Chinese National Medical Products Administration (NMPA).

Coblopasvir (formerly coded as KW‐136) is a pangenotypic inhibitor against HCV non‐structural protein (NS) 5A with picomolar antiviral activities against HCV replicons or cell culture systems of genotypes 1a, 1b, 2a, 3a, 4a, 5a and 6a in vitro (data on file). Coblopasvir demonstrates an additive or synergic effect when combined with interferon, NS3/4A protease inhibitor or NS5B nucleotide analogue, with no detected cross‐resistance with protease inhibitors or nucleotide analogues in vitro (data on file). In early‐phase clinical pharmacology studies, oral coblopasvir shows a favourable pharmacokinetics and tolerability profile in healthy participants, enabling a further efficacy proof‐of‐concept study, in which a maximal HCV ribonucleic acid (RNA) reduction of up to 5log_10_ IU/mL was observed in non‐cirrhotic patients of genotype 1b receiving an ultrashort‐duration (72‐hour) monotherapy (unpublished data). In a previous phase 2 study, a standard 12‐week treatment regimen of coblopasvir 30 or 60 mg with sofosbuvir 400 mg resulted in a sustained virological response (SVR) of 98% among treatment‐naïve Chinese patients infected with HCV of genotypes 1, 2, 3 and 6, including those with compensated cirrhosis.^4^

The primary objective of this phase 3 study was to evaluate the efficacy and safety of a 12‐week combo regimen of coblopasvir 60 mg plus sofosbuvir 400 mg for Chinese adult patients chronically monoinfected with HCV of diverse genotypes, including those with compensated cirrhosis and those having previously experienced interferons. We also analysed the possible confounding effects of HCV genotype, liver fibrosis and interferon experience on SVR.

## 2 METHODS

### 2.1 Study protocol and participants

This single‐arm, open‐label, phase 3 study was conducted at 19 clinical sites across China. The study protocol was approved by the Institutional Review Board or Independent Ethics Committee at each participating site, and the study was conducted in accordance with *the Declaration of Helsinki*, *the International Conference on Harmonization Good Clinical Practice* and other applicable national regulations. All participants volunteered to provide informed consent in writing before any study procedures.

The eligibility criteria were as follows: men and non‐pregnant and non‐lactating women aged 18‐70 years (inclusive); with documented chronic HCV monoinfection of genotypes 1‐6 or any other (sub)types, including mixed and indeterminate types; with a central laboratory confirmed plasma HCV RNA titre ≥10 000 IU/mL on screening; without cirrhosis or with evidenced compensated cirrhosis on precedent liver biopsy (F4 on Ishak, Metavir or GS scoring system) and/or liver transient elastography (FibroScan liver stiffness modulus [LSM] ≥14.6 kPa). Patients who had been previously exposed to interferons at least 6 months before screening could be enrolled, but those previously exposed to DAAs of any sources were excluded. Patients with unstable or uncontrolled medical conditions or co‐infected with hepatitis B virus (HBV) or human immunodeficient virus (HIV) were also excluded. Detailed inclusion and exclusion criteria are shown in Table S1 and the definitions of liver fibrosis are shown in Table S2.

### 2.2 Procedures

Patients were instructed to self‐administer coblopasvir capsules 60 mg and sofosbuvir tablets 400 mg (Kawin Technology Share‐Holding Co., Ltd., Beijing, China) with or without meal, once daily for 12 successive weeks. No dose modification was allowed throughout the treatment period.

Efficacy and safety were continuously monitored at treatment weeks 1, 2, 4, 8 and 12, and at post‐treatment weeks 4 and 12. Consenting patients entered into an optional extended follow‐up study at post‐treatment week 24 for the assessment of SVR durability (SVR24). The HCV RNA titre was quantitated using the COBAS AmpliPrep/COBAS Taqman HCV Test version 2.0 Virus Quantitative Detection Kit (Roche Molecular Diagnostics, Indianapolis, IN, USA) with a lower limit of quantitation (LLOQ) of 15 IU/mL and an upper limit of quantitation of 10^8^ IU/mL. The HCV genotype and subtype were sequenced using the reverse transcription polymerase chain reaction test (the Sanger method; HCV RNA ≥10^4^ IU/mL with a sensitivity of 20%). HCV RNA quantitation, HCV genotyping and HBV surface antigen (HBsAg) testing were conducted at a College of American Pathologists‐accredited central laboratory (Kingmed Center for Clinical Laboratory, Guangzhou, China), and other screening and safety laboratory tests were done at the local hospital clinical laboratory.

Pre‐existing and treatment‐emergent resistance‐associated substitutions (RASs) for genotypes 1b and 2a were tested using the population‐based sequencing technique at Kingmed (threshold ≥20% of a viral population) for NS5A and NS5B regions in plasma samples with an HCV RNA titre ≥1000 IU/mL from patients who experienced virological failure (including on‐treatment virological breakthrough, post‐treatment relapse, premature withdrawal and loss to follow‐up) compared to those collected at screening.

Safety was monitored at every study visit until post‐treatment week 12. Safety measures included adverse events (AEs), vital signs, physical examination, clinical laboratory tests, electrocardiography and upper abdominal ultrasonography. AEs were coded using *the Medical Dictionary for Regulatory Activities (MedDRA), version 20.0* (MedDRA MSSO, McLean, VA, USA) and graded using *the National Cancer Institute Common Terminology Criteria for Adverse Events (CTCAE), version 4.0*. The attribution of causality for any AE to the study drug was at the discretion of the investigator according to a national adverse drug reaction (ADR) vigilance procedure. ADR is defined as any AE definitely, probably, or possibly caused by use of the study drug, as assessed by the investigator.

### 2.3 Outcome measures

Efficacy and safety were assessed in all patients receiving at least one dose of the study drug. The primary efficacy endpoint was SVR12, defined as the proportion of patients with virological response (HCV RNA titre below LLOQ or target not detected) at 12 weeks after the completion or discontinuation of treatment. The secondary efficacy endpoints included the proportions of patients who achieved virological response at treatment weeks 1, 2, 4, 8 and 12 and at post‐treatment week 4, the proportion of patients who experienced on‐treatment virological breakthrough at treatment weeks 2, 4, 8 and 12 and the proportions of patients who experienced post‐treatment virological relapse at post‐treatment weeks 4 and 12. The exploratory efficacy endpoint was SVR24, defined as the proportion of patients who achieved SVR at 24 weeks after the completion of treatment among those who achieved SVR12 and completed the post‐treatment week 24 visit. Safety endpoints included AE, serious AE, vital signs, physical examination, clinical laboratory tests, 12‐lead electrocardiography and other safety tests. Detailed definitions of the virological responses are shown in Table S3.

### 2.4 Sample size estimation and statistical analysis

The sample size was estimated based on a superiority hypothesis test. For the FAS, the overall SVR12 for patients receiving coblopasvir plus sofosbuvir was conservatively estimated at 90% and that for a historical control was set at 85%^3^ in communication with the regulatory agency. A sample size of 324 patients would provide a statistical power of 85% at a one‐sided significance level of 0.025. In consideration of enrolling an adequate number of patients with genotypes 3 and 6, the sample size was set at 360 patients; genotype distribution was also set as follows to represent the real‐world HCV genotype profile in China^2^: genotype 1 and others at 50%, genotype 2 at 25%, genotype 3 at 12.5% and genotype 6 at 12.5%. The proportion of patients with advanced fibrosis (F3) or compensated cirrhosis (F4) was capped at 20%, and that of interferon‐experienced patients was also capped at 10%.

Point estimates and two‐sided 95% confidence intervals (95% CIs) were calculated using the Clopper‐Pearson method for primary and secondary efficacy endpoints. Missing HCV RNA data for any reason were counted as treatment failure for the full analysis set (FAS) using the intention‐to‐treat principle. Exploratory efficacy endpoints and safety endpoints were descriptively summarized. The SVR of patient subsets and the potential effects of genotype, liver fibrosis and interferon experience on SVR (expressed as odds ratio [OR] and 95% CI) were analysed in a *post hoc* manner using the logistic regression model with bootstrapping. All statistical summaries and analyses were performed using the SAS software package version 9.4 (SAS Institute Inc, Cary, NC, USA).

This trial is registered with ClinicalTrials.gov, number NCT03995485, and with ChinaDrugTrials.org.cn, number CTR20171654.

## 3 RESULTS

### 3.1 Patient characteristics

**FIGURE 1:**
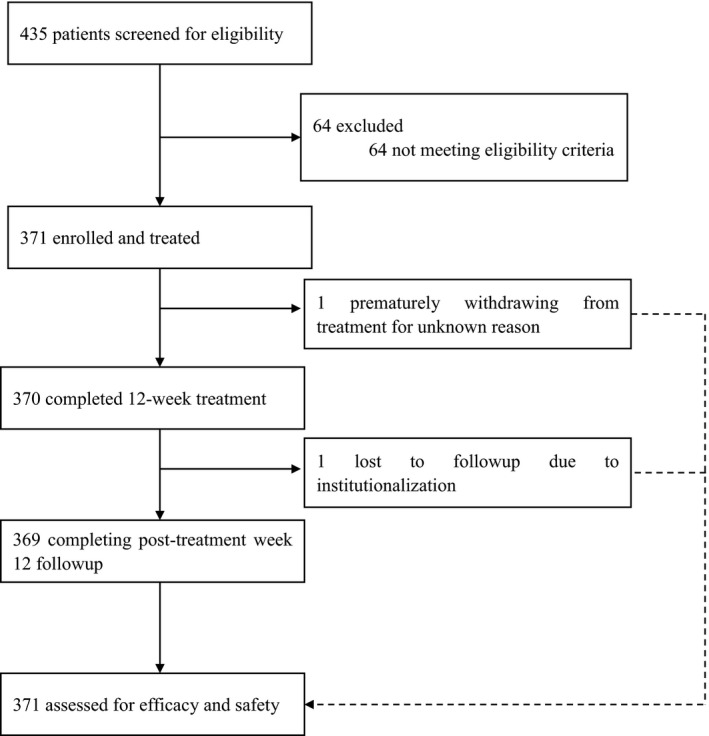
Study flow chart

Between June and August, 2017, 435 patients were screened, 64 of whom were excluded mainly as a result of not meeting the eligibility criteria for laboratory tests. Overall, 371 patients were enrolled in this study and treated with coblopasvir plus sofosbuvir. All patients completed the 12‐week treatment and additional 12‐week follow‐up visits, except for one patient prematurely withdrawn from treatment at week 2 for unknown reasons and another patient lost to post‐treatment week 12 follow‐up after completion of treatment as a result of institutionalized drug abstinence (Figure 1).

Overall, the study population consisted of a similar proportion of men and women (51% vs 49%), with a median age of 49 years (range, 19‐69 years) and a body mass index of 18 ~ 32 kg/m^2^, the majority of whom were Han Chinese (80%) in ethnicity (Table 1). All patients were seronegative to HBV and HIV. The genotype distribution was as follows (n = 371): genotype 1a, <1% (n = 2); 1b, 48% (n = 178); 2a, 26% (n = 95); 3a, 6% (n = 23); 3b, 7% (n = 27); 6, 12% (n = 46); no genotype 4 or 5 was detected or enrolled. Fifty‐one patients (n = 51, 14%) had F3 fibrosis and 39 patients (11%) had F4 fibrosis (compensated cirrhosis). Thirty‐nine patients (n = 39, 11%) had been previously exposed to interferons, most of whom had virological relapse or intolerance. None of the patients had a serum creatinine clearance below 50 ml/min (using the Cockcroft‐Gault formula) per the eligibility criteria. The most often reported concomitant medical conditions were non‐alcoholic fatty liver disease and essential hypertension.

**TABLE 1:** Patient demographics and baseline characteristics

	Patients (n = 371)
Age, years, median (range)	49 (19‐69)
Gender	
Male	190 (51%)
Female	181 (49%)
Ethnicity	
Han Chinese	295 (80%)
Others	76 (20%)
Body mass index, kg/m^2^, median (range)	24 (18‐32)
HCV genotype	
1	180 (49%)
1a	2 (<1%)
1b	178 (48%)
2	95 (26%)
2a	95 (26%)
3	50 (13%)
3a	23 (6%)
3b	27 (7%)
6	46 (12%)
6a	41 (11%)
6e	3 (<1%)
6n	2 (<1%)
Others	0 (0%)
HCV RNA titre, IU/mL, median (range)	1,760,000 (10,000‐18,800,000)
Liver fibrosis	
F0‐2	281 (76%)
F3	51 (14%)
F4^a^	39 (11%)
Previous interferon experience	
No	332 (89%)
Yes	39 (11%)
Non‐responder	4 (1%)
Breakthrough	2 (<1%)
Relapse	17 (5%)
Intolerance	15 (4%)
Serum creatinine clearance, ml/min, median (range)	102 (50‐226)
Concomitant medical conditions (≥10%)	
Fatty liver disease	52 (14%)
Essential hypertension	54 (15%)

^a^ All with compensated cirrhosis (Child‐Pugh class A). Data are in n (%) unless otherwise specified. HCV, hepatitis C virus; RNA, ribonucleic acid.

### 3.2 Virological responses

All compliant patients achieved a virological response by treatment week 8. Detailed on‐treatment virological responses are shown in Table S4. Among the 371 patients enrolled, 359 patients (97%; 95% CI [95%, 99%]) achieved the primary efficacy endpoint of SVR12 (Table 2). This high SVR12 was significantly greater than the prespecified 85% performance goal (*P* < .001), meeting the primary efficacy endpoint for this study. Per protocol set analysis also showed a similar result (358/368, 97%; 95% CI [96%, 99%], *P* < .001). Three hundred and fifty‐one patients (n = 351) who achieved SVR12 completed the post‐treatment week 24 visit, all of whom achieved SVR24 with the exception of one patient, representing a consistence of >99% between SVR24 and SVR12.

**TABLE 2:** SVR12 by genotype, fibrosis and interferon experience for full analysis set (n = 371)

SVR12	Overall (n = 371)
Overall	97% (359/371) [94%, 98%]
By genotype	
Genotype 1 (n = 180)	99% (178/180) [96%, >99%]
Genotype 2 (n = 95)	96% (91/95) [90%, 99%]
Genotype 3 (n = 50)	90% (45/50) [78%, 97%]
Genotype 3a (n = 23)	91% (21/23) [72%, 99%]
Genotype 3b (n = 27)	89% (24/27) [71%, 98%]
Genotype 6 (n = 46)	98% (45/46) [88%, >99%]
By fibrosis	
F0‐2 (n = 281)	97% (272/281) [94%, 99%]
F3 (n = 281)	98% (50/51) [90%, >99%]
F4 (n = 281)	95% (37/39) [83%, >99%]
By interferon experience	
Naïve (n = 332)	96% (320/332) [94%, 98%]
Experienced (n = 39)	**100% (39/39)** [91%, 100%]

Note

Data are in % (n/N) [95% confidence interval] using the Clopper‐Pearson method. ND, not done.

Subset analysis by genotype (Table 2) showed that patients with genotype 1 had the highest SVR12 (99%, 95% CI [96%, >99%]), compared to 96% (95% CI [90%, 99%]) for those with genotype 2, 90% (95% CI [78%, 97%]) for those with genotype 3 and 98% (95% CI [88%, >99%]) for those with genotype 6 respectively. *Post hoc* subset analysis of genotype 3 showed a similar SVR12 between subtypes 3a and 3b (91% [21/23] vs 89% [24/27]). Further sensitivity analysis for patients with genotype 3 showed an SVR12 of 96% (45/47; 95% CI [85%, >99%]) with three non‐compliant patients excluded. A high SVR12 was also observed among patients with fibrosis of variable severity (Table 2), 97% (95% CI [94%, 99%]) for F0‐2, 98% (95% CI [90%, >99%]) for F3 and 95% (95% CI [83%, >99%]) for F4 respectively. Interferon‐experienced patients achieved a SVR12 of 100% (95% CI [91%, 100%]) (Table 2).

Univariate analysis showed that genotypes 3a (odds ratio [OR]=8.48 [1.13, 63.4], *P* = .002) and 3b (OR = 11.1 [1.77, 70.0], *P* < .001) were associated with a lower SVR12 compared to genotype 1, while fibrosis stages F3 (OR = 0.60 [0.08, 4.88], *P* = .296) and F4 (OR = 1.63 [0.34, 7.85], *P* = .424) did not significantly affect SVR12 compared to F0‐2 (Tables S5 and S6). Further multivariate analysis showed no interactive effect between genotype and fibrosis stage (*P* = .646). Adjustment of fibrosis slightly increased the SVR12 OR for genotype 3a (unadjusted OR = 8.48 [1.13, 63.4], *P* = .002; adjusted OR = 9.01 [1.20, 67.9], *P* < .001) or 3b (unadjusted OR = 11.1 [1.77, 70.0], *P* < .001; adjusted OR = 12.0 [1.88, 76.6], *P* < .001) compared to genotype 1, while adjustment of genotype did not significantly affect the SVR12 OR for fibrosis staging (Table S6). No univariate or multivariate analysis was performed for interferon treatment experience as interferon‐experienced patients achieved a SVR12 of 100%.

### 3.3 Virological failure

Of 371 patients, 12 patients (3%) did not achieve SVR12 (Table 3), all of whom were naïve to interferon treatment. Ten patients (n = 10, 3%) experienced virological relapse, including two patients (n = 2) with genotype 1 and with F0‐2 or F4, four patients (n = 4) with genotype 2a and with F0‐2, three patients (n = 3) with genotype 3 and with F0‐2 (genotype 3a, voluntary interruption of self‐dosing between treatment weeks 2 and 4 followed by virological breakthrough at treatment week 4), F3 or F4 (both of genotype 3b), and one patient (n = 1) with genotype 6n with F0‐2.

**TABLE 3:** Virological failures for full analysis set (n = 371)

	Patients
Virological failures^a^	12 (3%)
Virological relapse	10 (3%)
At post‐treatment week 4	8 (2%)
at post‐treatment week 12	2 (<1%)
Virological breakthrough^b^	0 (0%)
Lost to follow‐up and others^c^	2 (<1%)

^a^ Defined as not achieving SVR12 (sustained virological response at post‐treatment week 12).

^b^ One compliant patient of genotype 3b with F3 experienced breakthrough at treatment week 2 but achieved SVR12.

^c^ Including one patient of genotype 3b with F0‐2 who prematurely withdrew from treatment at week 2 for unknown reasons and one patient of genotype 3a with F0‐2 lost to follow‐up at post‐treatment week 12 as a result of institutionalization. Data are in n (%).

One treatment‐naïve patient with genotype 1b and with F0‐2 experienced virological relapse at post‐treatment week 4 but achieved SVR12. One treatment‐naïve patient with genotype 6e with F0‐2 achieved SVR12 but relapsed at post‐treatment week 24.

One compliant patient of genotype 3b with F3 experienced virological breakthrough at treatment week 2 (87 IU/mL) from <15 IU/mL at treatment week 1 but achieved SVR12. One patient (genotype 3b with F0‐2) prematurely withdrew from the study after completing 2‐week treatment for unknown reasons, and one patient (genotype 3a with F0‐2) was lost to follow‐up as a result of institutionalization at post‐treatment week 12.

All of these patients achieved virological response at the time of treatment completion, withdrawal or the last visit before they were lost to follow‐up. A detailed description of virological failure is shown in Table S7.

### 3.4 Resistance monitoring

Fifteen patients (n = 15) were eligible for predefined polymorphism sequencing for NS5A and NS5B. Polymorphism sequencing was performed for six patients (n = 6) with genotype 1b (n = 2) or 2a (n = 4), but not for nine patients (n = 9) with genotype 1a (n = 1), 3a (n = 2), 3b (n = 4), 6e (n = 1) and 6n (n = 1) as a result of unavailability of subtype‐specific polymorphism sequencing methodology at the time of conducting this study. Among six patients (n = 6) with NS5A polymorphism data available, the common pre‐existing RAS included Y93H for NS5A of genotype 1b (n = 2) and L31M for NS5A of genotype 2a (n = 4), and no treatment‐emergent NS5A polymorphism was detected. No pre‐existing or treatment‐emergent S282T, the major NS5B RAS, was detected in these six patients (n = 6) with NS5B polymorphism data available. A detailed description of the NS5A and NS5B polymorphisms is shown in Table S7.

### 3.5 Safety data

Treatment‐emergent AEs (TEAEs) were reported for 292 patients (79%), comprising 193 patients with grade 1 (52%), 86 patients with grade 2 (23%), 11 patients with grade 3 (3%) and 2 patients with grade 4 (<1%) (Table 4). Grade 4 AEs were acute pancreatitis and hypertensive crisis, which resolved after in‐hospital symptomatic treatment. None of the grade 3 and 4 AEs were judged by the investigators to be associated with use of the study drug. No patients discontinued or interrupted treatment because of AEs.

**TABLE 4:** Adverse events and laboratory abnormalities

	Patients (n = 371)
Any TEAEs	292 (79%)
Grade 3	11 (3%)
Grade 4	2 (<1%)
Any serious AEs	12 (3%)
Any AEs leading to discontinuation of study drug	0 (0%)
Death	0 (0%)
Any TEAE‐related study drug	102 (27%)
Grade 1	83 (22%)
Grade 2	19 (5%)
Grade 3 or 4	0 (0%)
Any TEAEs or TEAE‐related study drug ≥ 5%	0 (0%)
Any TEAE related to study drug ≥ 1%	
Fatigue	10 (3%)
Headache	7 (2%)
Dizziness	6 (2%)
Diarrhoea	6 (2%)
Nausea	4 (1%)
Abdominal pain	4 (1%)
Lethargy	4 (1%)
Fatty liver	4 (1%)
Grade 3 or 4 laboratory abnormalities of clinical significance	0 (0%)
Laboratory abnormalities ≥1%	
Neutropenia	14 (4%)
Hypoalbuminemia	10 (3%)
Hyperuricemia	6 (2%)
Thrombocytopenia	5 (1%)

Note

Data are n (%). AE, adverse events; TEAEs, treatment‐emergent adverse events.

One hundred and two patients (n = 102, 27%) experienced TEAEs related to study drug, including 83 patients with grade 1 (22%) and 19 patients with grade 2 (5%); none of the patients experienced grade 3 or 4 TEAEs related to study drug. No AEs or TEAEs related to study drug were reported at a frequency ≥5%, and the most often reported TEAEs related to study drug (≥1%, excluding laboratory abnormalities) were fatigue (3%), headache (2%), dizziness (2%), diarrhoea (2%), nausea (1%), abdominal pain (1%), lethargy (1%) and fatty liver (1%). The majority of AEs and TEAEs related to study drug were transient and required no specific medical intervention.

Twelve patients (n = 12, 3%) experienced serious AEs, which were mainly hospitalizations as a result of elective or emergency operations. None of the serious AEs were judged to be related to the study drug. No deaths occurred.

No grade 3 or 4 laboratory abnormalities of clinical significance were reported. The most often reported laboratory abnormalities (≥1%) were neutropenia (4%), hypoalbuminemia (3%), hyperuricemia (2%) and thrombocytopenia (1%). No clinically significant, non‐isolated worsening was reported for haematology, urinalysis, clinical biochemistry or coagulation. General liver function tests, including alanine aminotransferase, aspartate aminotransferase and gamma‐glutamyl transferase, showed a significant trend of normalization throughout the study period (Figure S1A‐C). FibroScan also showed a trend in improved LSM, especially for patients with F3 or F4 (Figure S1D). Isolated, elevated creatinine was reported for three patients (n = 3) and assessed to be not clinically significant.

Of two patients (n = 2) with increased alfa‐fetal protein, one patient with genotype 1b and with F4 relapsed at post‐treatment week 12 (HCV RNA titre approximating 100 IU/mL) and was further diagnosed with hepatocellular carcinoma on increased serum alfa‐fetal protein combined with contrast liver imaging. One patient had a prolonged QT interval on treatment, which resolved without medical intervention 1 month later.

## 4 DISCUSSION

Our study population was highly representative of Chinese real‐world patients infected with HCV^2^ and comparable to that reported for the China phase 3 study of velpatasvir‐sofosbuvir in terms of age, gender, genotype (also no genotype 4 or 5 detected), HCV titre, fibrosis and previous treatment history.^3^ After a universal, standard 12‐week, fixed‐dose combo treatment with coblopasvir plus sofosbuvir, the SVR12 was 97% (359/371) for the patients overall and above 95% for patients with genotypes 1, 2 and 6. The SVR for patients with genotype 3 was slightly lower but still at 90% (45/50). The slightly lower virological response was primarily driven by three patients with poor on‐treatment or post‐treatment follow‐up compliance; with the poorly compliant patients excluded, SVR12 was achieved in 96% (45/47) of patients with genotype 3. SVR12 was also high for patients with compensated cirrhosis (95%) and was achieved by 100% of interferon‐experienced patients. The high SVR after treatment with coblopasvir plus sofosbuvir showed no significant effect confounded by the HCV genotype, baseline fibrosis, previous interferon exposure or the interactive effects of these factors.

Together with the rest of the world, China aims to achieve ‘No HepC’ by the year 2030, requiring at least 80% of patients cured by that year.^5^ Therefore, a simple‐to‐use, highly effective and publicly affordable pangenotypic treatment regimen is mandatory to achieve this public health goal. The combo regimen of coblopasvir plus sofosbuvir requires no sophisticated pre‐treatment genotyping or baseline liver fibrosis assessment and, therefore, enables the delivery of care to patients in the setting of real‐world general practice. Use of this domestic‐made combo regimen as a first‐line, general purpose candidate is expected to be cost saving and meet the ‘unmet medical needs’ of HCV‐infected patients in China.^6^

SVR12 after 12‐week treatment with coblopasvir plus sofosbuvir (97%) was generally similar to that with velpatasvir‐sofosbuvir for Chinese patients (96%) (Table S8).^3^ As Wei *et al* ^3^ reported a lower efficacy (76%) of velpatasvir‐sofosbuvir in Chinese patients with genotype 3b, a *post hoc* genotype 3 subtype SVR analysis was conducted, showing a comparable response rate between patients with genotypes 3a and 3b (91% [21/23] vs 89% [24/27]) in our study. However, a further analysis with non‐compliant patients excluded showed a higher SVR for patients with genotype 3a (100% [21/21]) compared to that for genotype 3b patients (92% [24/26]). The SVR12 for genotype 3 with coblopasvir plus sofosbuvir (90% [45/50]) was also noted to be greater than that with velpatasvir‐sofosbuvir (83% [49/59]). With non‐compliant patients excluded, the difference in the response rate was even greater (96% [45/47] vs 84% [49/58]). Further subtype analysis revealed this difference was primarily driven by that for genotype 3b (FAS, 89% [24/27] *vs* 78% [29/37]; compliant, 92% [24/26] vs 78% [29/37]) rather than genotype 3a (FAS, 91% [21/23] vs 91% [20/22]; compliant, 100% [21/21] vs 95% [20/21]). This finding should be cautiously interpreted as only three patients (n = 3) with genotype 3b with cirrhosis were enrolled in this study compared to 14 patients in the China phase 3 study of velpatasvir‐sofosbuvir.^3^ The actual response of cirrhotic patients with genotype 3b to coblopasvir plus sofosbuvir requires more clinical data from post‐marketing real‐world studies. However, the public health effect of a lower response for patients with genotype 3b, especially in cirrhotic patients, is expected to be minimal from the perspective of ‘No HepC’ as the prevalence of this subpopulation accounts for only 0.7% in China.^7^

Two patients (n = 2) with genotypes 6e and 6n experienced post‐treatment relapse. These two less common subtypes were not evaluated in the China phase 3 study of velpatasvir‐sofosbuvir,^3^ but is relatively more common in Thai patients (predominance accounting for 1% and 22% of genotype 6, respectively)^8^ and was also detected in Chinese injection drug users (predominance accounting for 9% and 3% of injection drug users respectively).^9^ The treatment efficacy of coblopasvir, along with other pangenotypic NS5A inhibitors, plus sofosbuvir warrants further evaluation in this special population infected with HCV of profound genetic diversity.

Owing to the small number (n = 5) of patients with genotype 1b (n = 1, with F4) or 2a (n = 4, with F0‐2) who failed treatment with coblopasvir plus sofosbuvir, the effect of pre‐existing and/or treatment‐emergent RASs for NS5A or NS5B on the virological response could not be analysed for patients with genotype 1b or 2a; however, no treatment‐emergent RAS was detected in these five patients. In the previous efficacy proof‐of‐concept study of an ultrashort‐duration (72‐hour) coblopasvir monotherapy, pre‐existing RASs for NS5A were detected in 31/36 (86%) of non‐cirrhotic patients with genotype 1b (18/22 [82%]; >10%, R30Q[5/22, 23%], Q54H, Q54Y or Q54Q/H [6/22, 27%], P58Q or P58S [3/22, 17%], Q62H, Q62L, Q62N, Q62R or Q62S [6/22, 27%], A92T or A92A/T [4/22, 18%] and Y93H or Y93Y/H [5/22, 23%]) or 2a (13/14, 93%; L31M, 13/14 [93%]), consistent with a previous study with velpatasvir‐sofosbuvir in Chinese patients.^3^ Major treatment‐emergent NS5A polymorphisms included L31M, L31V and Y93H for genotype 1b following 72‐hour coblopasvir monotherapy, and RAS L31M was persistent for genotype 2a until 216 hours after the first doing of coblopasvir (unpublished data). In another independent study conducted by the central laboratory Kingmed, Y93H was frequently (14.1%) detected in DAA‐naïve patients with genotype 1b, and L31M was also prevalent (95.6%) among naïve patients with genotype 2a.^10^ Therefore, pre‐existing NS5A polymorphisms might also be prevalent in the patients with genotype 1b and 2a in this study, although NS5A polymorphisms were not genotyped in patients with genotype 1b or 2a who achieved SVR12. Together with the RAS data for NS5A in the China phase 3 study of velpatasvir‐sofosbuvir,^3^ it can be expected that baseline NS5A polymorphisms have no significant effect on the SVR of patients with genotype 1b or 2a following treatment with coblopasvir plus sofosbuvir.

The prevalence and response effect of NS5A polymorphisms remain unknown for patients with genotypes 1a, 3a, 3b and 6 in this study. In the independent study by Kingmed, pre‐existing NS5A RASs were less common in naïve patients with genotype 3a (Y93H, 3.3%) but highly prevalent in those with genotype 3b, with 97.4% for A30K and 98.7% for L31M, which resulted in a 96% presence of double mutation A30K + L31M.^10^ It has been reported that the baseline RASs for NS5A does not affect SVR12 in the majority of these subtypes; however, RASs for NS5A were pre‐existent in all patients with genotype 3b, mainly because of the combination of A30L and L31M conferring a high‐level (>100‐fold) resistance to velpatasvir.^3^ Pretreatment resistance screening is not generally recommended, while known cirrhotic patients of genotype 3b may need a more potent regimen than the standardized ribavirin‐free 12‐week treatment with a pangenotypic NS5A inhibitor plus sofosbuvir. The treatment regimen options currently available include the addition of ribavirin and/or doubled course (ie 24‐week treatment).^11^, ^12^ Use of sofosbuvir‐velpatasvir‐voxilaprevir was approved only for patients with genotype 3 previously exposed to sofosbuvir or an NS5A inhibitor,^13^ the effectiveness of which remains questionable for patients exposed to both sofosbuvir and NS5A inhibitors. This triple‐DDA regimen cannot be recommended as the first‐line treatment of option for possibly refractory patients with genotype 3b with cirrhosis. It may remain an ‘unmet medical need’ for this small population of patients refractory to the DAA‐based regimens currently available.

The 12‐week treatment with coblopasvir plus sofosbuvir showed a generally favourable safety and tolerability profile, consistent with those reported in previous studies of ribavirin‐free, all‐oral DAA regimens. Of note, patients showed a trend in liver disease improvement with respect to liver inflammation and fibrosis, with a generally stable renal function profile, throughout the on‐treatment and post‐treatment follow‐up periods. Treatment‐emergent HCC has been reported and is considered a predictor of virological relapse.^14^ The beneficial effect of DAA regimens is evident from the reduced occurrence of HCC but liver malignancy should be monitored, especially for cirrhotic patients.^15^

The proportions of HCV genotype, liver fibrosis and previous interferon exposure were capped from the regulatory perspective, although the study population maximally represented the real‐world population of HCV‐infected patients in China. Therefore, the generalization of the results of this study should be further validated in post‐marketing studies and observations in Chinese patients with highly diversified baseline characteristics, especially for these special populations.

In conclusion, the ribavirin‐free, all‐oral, pangenotypic combo regimen of coblopasvir plus sofosbuvir demonstrates a high SVR and a favourable safety profile for Chinese adult patients chronically monoinfected with HCV, including those with compensated cirrhosis. This regimen requires no pretreatment assessment of HCV genotype or liver fibrosis, and the treatment duration is fixed at 12 weeks for all patients regardless of the baseline characteristics. These clinical benefits and the affordability of this combo regimen address the ‘unmet medical needs’ for chronic hepatitis C in China and facilitate the goal of a ‘No HepC’ China by the year 2030.

## CONFLICT OF INTEREST

Jing Ning, Hai Pan and Hong Qin were employees of Kawin Technology when executing this trial, and Li Li and Desheng Zhou are employees and stakeholders of Kawin Technology. The other authors have no conflict of interest to declare.

## Supporting information

Supplementary Material
