# Supplementary material for: Coblopasvir and sofosbuvir for treatment of chronic hepatitis C virus infection in China: A single‐arm, open‐label, phase 3 trial
Source: Liver Int. 2020 Oct 13;40(11):2685–93. doi: 10.1111/liv.14633 (PMC7702130; doi:10.1111/liv.14633)
Supplement: Supplementary file 1 — Supplementary Material [file LIV-40-2685-s001.docx]

**Coblopasvir and sofosbuvir for treatment of chronic hepatitis C virus infection in China: a single-arm, open-label, phase 3 trial**

**Short title:** Coblopasvir and sofosbuvir for chronic hepatitis C

Yanhang Gao^1^, Fei Kong^1^, Guangming Li^2^, Cheng Li^2^, Sujun Zheng^3^, Jianmei Lin^4^, Xiaofeng Wen^5^, Jinghua Hu^6^, Xiaozhong Wang^7^, Xiaofeng Wu^8^, Huichun Xing^9^, Jidong Jia^10^, Zhansheng Jia^11^, Yujuan Guan^12^, Chenghao Li^13^, Guicheng Wu^14^, Zhiliang Gao^15^, Zhuangbo Mou^16^, Qin Ning^17^, Qing Mao^18^, Yongfeng Yang^19^, Jing Ning^20^, Li Li^20^, Hai Pan^20^, Desheng Zhou^20^,Yanhua Ding^21,*^, Hong Qin^20,*^, Junqi Niu^1,*^

1. Department of Hepatology, the First Hospital of Jilin University, Changchun, Jilin, China
2. Cirrhosis Department, Zhengzhou Sixth Municipal People’s Hospital, Zhengzhou, Henan, China
3. Difficult & Complicated Liver Diseases and Artificial Liver Center, Beijing You An Hospital, Capital Medical University, Beijing, China
4. Department of Infectious Diseases, Sichuan Provincial People’s Hospital, Chengdu, Sichuan, China
5. Department of Hepatology, Liuzhou People’s Hospital, Liuzhou, Guangxi, China
6. Liver Failure Treatment and Research Center, the Fifth Medical Center of PLA General Hospital , Beijing, China
7. Department of Hepatology, Xinjiang Uygur Autonomous Region Traditional Chinese Medicine Hospital, Urumqi, Xinjiang, China
8. Department of Hepatology, Shenyang Sixth People’s Hospital, Shenyang, Liaoning, China
9. Department of Hepatology Division 3, Beijing Ditan Hospital, Capital Medical University, Beijing, China
10. Liver Research Center, Beijing Youyi Hospital Affiliated to Capital Medical University, Beijing, China
11. Department of Infectious Diseases, the Second Affiliated Hospital of People’s Liberation Army Air Force Medical University, Xi’an, Shaanxi, China
12. Department of Hepatology, Guangzhou Eighth People’s Hospital, Guangzhou, Guangdong, China
13. Department of Gastroenterology, Yanbian University Affiliated Hospital, Yanji, Jilin, China
14. Department of Hepatology, Chongqing University Three Gorges Hosipital, Chongqing Three Gorges Central Hospital, Wanzhou, Chongqing, China
15. Department of Infectious Diseases, the Third Affiliated Hospital of Dr Sun Yat-Sen University, Guangzhou, Guangdong, China
16. Department of Hepatology, Ji’nan Municipal Hospital of Infectious Diseases, Ji’nan, Shandong, China
17. Department of Infectious Diseases, Tongji Hospital Affiliated to Tongji Medical College, Huazhong University of Science and Technology, Wuhan, Hubei, China
18. Institute of Infectious Diseases, the First Affiliated Hospital of People’s Liberation Army Medical University, Chongqing, China
19. Department of Hepatology, Nanjing Second Municipal Hospital, Nanjing, Jiangsu, China
20. Research and Development Center, Beijing Kawin Technology Share-Holding Co., Ltd., Beijing, China
21. The Department of Phase I Clinical Trial, the First Hospital of Jilin University, Changchun, Jilin, China

*Co-primary authors.

**Correspondence to:**

Prof. Junqi Niu, Department of Hepatology, the First Hospital of Jilin University, No. 71 Xinmin Street, Changchun 130021, Jilin, China; Tel: +86 431 81875101; Fax: +86 431 81875101; Email: [junqiniu@aliyun.com](mailto:junqiniu@aliyun.com)

Dr. Hong Qin, Research and Development Center, Beijing Kawin Technology Share-Holding Co., Ltd., No. 6 Rongjing East Street, BDA, Beijing 100176, China

Tel: +86 10 8712 0895; Fax: +86 10 6787 2896; Email: [qinhong@kawin.com.cn](mailto:qinhong@kawin.com.cn)

Current work address: Clinical Development, Hangzhou Sciwind Biosciences Co., Ltd., Suite 815-818, Building No. 17, No. 57 Kejiyuan Avenue, Qiantang New District, Hangzhou 310026, Zhejiang, China; Tel: +86 136 79290113; Fax: +86 10 67872896; Email: qinhong@sciwind.com.cn

Prof. Yanhua Ding, The Department of Phase I Clinical Trial, the First Hospital of Jilin University, No. 71 Xinmin Street, Changchun 130021, Jilin, China; Tel: +86 431 88782705; Fax: +86 431 88782705; Email: dingyanhua2003@126.com

**SUPPLEMENTARY TABLES**

**Supplementary Table S1.** Inclusion and exclusion criteria.

**Supplementary Table S2.** Definitions of liver fibrosis.

**Supplementary Table S3.** Definitions of virologic response and interferon treatment experience.

**Supplementary Table S4.** Secondary efficacy endpoints-virologic responses.

**Supplementary Table S5.** SVR12 by genotype and fibrosis interaction for full analysis set (n=371).

**Supplementary Table S6.** Univariate and multivariate logistics regression analyses of genotype, fibrosis and interferon experience associated for FAS and compliant subset.

**Supplementary Table S7.** Virologic failures and baseline/treatment-emergent NS5A/NS5B polymorphisms.

**Supplementary Table S8.** SVR12 with coblopasvir plus sofosbuvir in pooled phase 2/3 studies *versus* with velpatasvir-sofosbuvir in China phase 3 study (not head-to-head).

**Supplementary Table S1.** Inclusion and exclusion criteria.

| **Study Title:** Evaluation of efficacy and safety of KW-136 capsule combined with sofosbuvir tablet for treatment of adult chronic hepatitis C: an open-label, multi-center, phase 3 study |
| --- |
| **Study Protocol Number:** KYGL-2017-001 |
| **ClinicalTrials.gov Identifier:** NCT03995485 |
| **ChinaDrugTrials.org.cn Identifier:** CTR20171654 |
| **Inclusion Criteria:** |
| Participants must meet **ALL** the following criteria to be enrolled.   - aged between 18 and 70 years (both inclusive) at the time of informed consenting and of either sex; - with a body mass index (BMI) between 18 and 32 kg/m^2^ (both inclusive); - chronically infected with hepatitis C virus (HCV), namely, with positive anti-HCV, HCV ribonuclear acid (HCV RNA) or genotyping results at least six (6) months before the screening, or with a liver biopsy confirming chronic hepatitis within twelve (12) months before the screening or within the screening period; - with anti-HCV positivity and at least once testing result with HCV RNA equaling to or above 10^4^ IU/mL within the screening period; - with genotype 1, 2, 3, 4, 5, 6, mixed, indeterminate or any other genotype as determined by the centralized laboratory genotyping; - no more than ten percent (10%) of the enrolled participants having previously experienced interferon (defined as having received any regulatory agency approved or investigational interferon formulations, including pegylated and regular interferons at least six (6) months before the screening); - having not previously experienced any other approved, investigational or unapproved direct antiviral agents against HCV of any source; - having not previously experienced oral or injective ribavirin within three (3) months before the screening; - no more than twenty percent (20%) of the enrolled patients having advanced fibrosis or compensatory cirrhosis; - women of childbearing potential (including postmenopausal women at or under 50 years of age) with negative blood pregnancy test results, and participants of childbearing potential (including male participants and their female partners) having no childbearing plan and consenting to voluntarily use effective contraceptive measures from the screening until six (6) months after the end of treatment; - lactating women consenting to discontinue nursing from the screening until six (6) months after the end of treatment; - voluntarily participating in this trial and being able to understand and sign the informed consent form. |
| **Exclusion Criteria:** |
| Participants must **NOT** meet **ANY** of the following criteria to be enrolled.   - having previously experienced any investigational or experimental direct antiviral agents against HCV, including protease inhibitor, nonstructural protein (NS) 5A inhibitor or NS5B polymerase inhibitor, before the screening; - having previously experienced interferon-based antiviral regimens within six (6) months before the screening; - having previously experienced oral or injective ribavirin within three (3) months before the screening; - having previously experienced any systemic potent immunomodulatory agents, such as steroids or thymosin alfa, excluding nasal, inhalational, topical steroids and/or others, for more than two (2) weeks within six (6) months before the screening, or expected to be exposed to these agents during the study period; - with hepatitis B virus surface antigen (HBsAg) or anti-human immunodeficient virus (HIV) positivity; - with evidence of decompensatory liver function, including but not limited to total serum bilirubin (TBIL) above twice (2) of the upper limit of normal (ULN), serum albumin (ALB) below 35 g/L or prothrombin activity (PTA) below 60% confirmed on repeated testing, previous or present history of ascites, upper gastrointestinal bleeding and/or hepatic encephalopathy, or with a liver function reserve of Child-Pugh class B or C; - with primary liver cancer confirmed or evidenced by serum alfa-fetoprotein (AFP) above 100 ng/ml or liver imaging study showing suspected nodules; - with a previous history of liver disease of any other cause, including alcoholic liver disease, nonalcoholic steatohepatitis, drug-induced hepatitis, autoimmune hepatitis, Wilson disease or hemochromatosis; - with serum alanine aminotransferase (ALT) or asparate aminotransferase (AST) above ten (10) times of the ULN confirmed on repeated testing; - with white blood cell (WBC) count below 3×10^9^ per liter, neutrophil count below 1.5×10^9^ per liter (or below 1.25×10^9^ per liter for cirrhotics), platelet count below 50×10^9^ per liter, or hemoglobin below 100 g/L confirmed on repeated testing; - with serum creatinine clearance (CLCr) below 50 ml/min using the Cockcroft-Gault formula confirmed on repeated testing; - with poorly controlled diabetes mellitus (hemoglobin A1c [HbA1c] above 8.0% confirmed on repeated testing); - with psychiatric or neurologic disorders, including previous or family history of psychiatric disorders (especially depression, depressive state, epilepsy or hysteria); - with serious cardiovascular disorders, including uncontrolled hypertension (systolic blood pressure at or above 160 mmHg and/or diastolic blood pressure at or above 100 mmHg), heart insufficiency of New York Heart Association class III or above, history of myocardial infarction within six (6) months before the screening, history of percutaneous transluminal coronary angioplasty within six (6) months before the screening, unstable angina pectoris, or QTc interval (Fridericia correction formula QTc = QT×RR^-1/3^) at or above 450 msec for males or 470 msec for females, second- or third-grade atrioventricular block or any other uncontrolled arrhythmias confirmed on repeated electrocardiography on screening; - with serious hematologic disorders, such as anemia, hemophilia and others; - with serious kidney diseases, such as chronic kidney disease, kidney insufficiency and others; - with serious gastrointestinal disorders, such as peptic ulcer, colitis and others; - with serious respirator disorders, such as active pulmonary tuberculosis, lung infection, chronic obstructive pulmonary disease, pulmonary interstitial disease and others; - with active or suspected malignant tumors, or with a previous history of malignant tumors, excluding skin basal cell carcinoma or cervical carcinoma *in situ*, within five (5) years before the screening; - with a history of major organ transplantation; - with a hypersensitive predisposition or a known history of serious allergy, especially to the investigational products and substances; - with a history of active alcohol or drug abuse within six (6) months before the screening - pregnant women or lactating women rejecting or unable to discontinue nursing; - unable to discontinue prohibited medications as defined by the protocol; - having previously participated in clinical studies of any other drugs within three (3) months before the screening; - unable or unwilling to provide informed consent, or unable to follow the protocol requirements; - with any other conditions of ineligibility at the discretion of the investigators. |

**Supplementary Table S2.** Definitions of liver fibrosis.

| **Liver fibrosis** | **Liver stiffness modulus (kPa) ^a^** | **Fibrosis staging ^b^** |
| --- | --- | --- |
| Without advanced fibrosis or cirrhosis | <9.6 | F0-2 |
| With advanced fibrosis | ≥9.6 but <14.6 | F3 |
| With cirrhosis | ≥14.6 | F4 |

^a^ on FibroScan at screening;

^b^ on liver biopsy as assessed using the Metavir, Ishak or GS scoring system within twelve (12) months before screening or at screening, with an evidence-level priority over FibroScan result and with the most recent biopsy result documented if multiple biopsy reports available.

**Supplementary Table S3.** Definitions of virologic response and interferon treatment experience.

| **Virologic response/**  **Interferon treatment experience** | **Definitions** |
| --- | --- |
| On-treatment response | HCV RNA titer below LLOQ or target not detected when on treatment |
| Sustained virologic response | HCV RNA titer below LLOQ or target not detected after completion or discontinuation of treatment |
| Breakthrough | On-treatment re-emergence of HCV RNA (equaling to or above LLOQ) for compliant patients having previously achieved on-treatment response |
| Relapse | HCV RNA titer equaling to or above LLOQ after completion or discontinuation of treatment for patients having achieved virologic response at treatment week 12 |
| Treatment-naïve (interferon) | Never been exposed to any injections of interferon (including both routine and pegylated formulations) |
| Nonresponse (interferon) | HCV RNA titer equaling to or above LLOQ after completion of a standard-course interferon treatment |
| Breakthrough (interferon) | On interferon treatment re-emergence of HCV RNA (equaling to or above LLOQ) for patients receiving a standard-course interferon treatment and having previously achieved on-treatment response |
| Relapse (interferon) | HCV RNA titer equaling to or above LLOQ after completion or discontinuation of treatment for patients having achieved virologic response at the end of a standard-course interferon treatment |
| Intolerance (interferon) | Unable to complete a standard-course interferon treatment due to any adverse events, including but not limited to any serious systemic or local adverse reactions (e.g., allergy, hypersensitiveness, and injection site reaction), neurologic or psychiatric disorders or any conditions requiring medical intervention, serious flu-like symptoms (e.g., fever, fatigue, myalgia, and arthralgia), serious gastrointestinal symptoms (e.g., nausea, vomiting, and diarrhea), serious hematologic disorders (e.g., anemia, neutropenia, and thrombocytopenia), colitis, pancreatitis, ocular fundus disorders, newly emergence or worsening of autoimmune disorders, or any other medically documented adverse events related or possibly related to interferon treatment |

**Supplementary Table S4.** Secondary efficacy endpoints-virologic responses.

| **Virologic response at** | **FAS (n=371)** | **PPS (n=368) ^*^** |
| --- | --- | --- |
| Treatment week 1 | 147 (40%) [35%, 45%] | 145 (39%) [34%, 44%] |
| Treatment week 2 | 299 (81%) [77%, 85%] | 298 (81%) [77%, 85%] |
| Treatment week 4 | 367 (99%) [98%, >99%] | 365 (99%) [98%, >99%] |
| Treatment week 8 | 370 (>99%) [99%, >99%] | 368 (100%) [100%, 100%] |
| Treatment week 12 | 369 (>99%) [99%, 100%] | 368 (100%) [100%, 100%] |
| Post-treatment week 4 | 361 (97%) [96%, 99%] | 359 (98%) [96%, 99%] |

Data are in n (%) [95% confidence intervals] using the Clopper-Pearson method.

^*^ Three patients (n=3) excluded from PPS due to premature withdrawal from treatment at week 2, missing on-treatment visit at week 12, or lost-to-followup at post-treatment week 12.

FAS, full analysis set; PPS, per protocol set.

**Supplementary Table S5.** SVR12 by genotype and fibrosis interaction for full analysis set (n=371).

| **SVR12** | **F0-2**  **(n=281)** | **F3**  **(n=51)** | **F-4**  **(n=39)** |
| --- | --- | --- | --- |
| Overall | 97% (272/281)  [94%, 99%] | 98% (50/51)  [90%, >99%] | 95% (37/39)  [83%, >99%] |
| Genotype 1 (n=180) | 99% (136/137)  [96%, >99%] | **100% (25/25)**  [86%, 100%] | 94% (17/18)  [73%, >99%] |
| Genotype 2 (n=95) | 95% (71/75)  [96%, 99%] | **100% (10/10)**  [69%, 100%] | **100% (10/10)**  [69%, 100%] |
| Genotype 3 (n=50) | 91% (30/33) ^a^  [76%, 98%] | 92% (11/12)  [62%, >99%] | 80% (4/5)  [ND]^b^ |
| *Genotype 3a (n=23)* | 88% (14/16)  [62%, 98%] | **100% (5/5)**  [ND] ^b^ | **100% (2/2)**  [ND] ^b^ |
| *Genotype 3b (n=27)* | 94% (16/17)  [71%, >99%] | 86% (6/7)  [ND] ^b^ | 67% (2/3)  [ND] ^b^ |
| Genotype 6 (n=46) | 97% (35/36)  [85%, >99%] | **100% (4/4)**  [ND] ^b^ | **100% (6/6)**  [ND] ^b^ |
| Interferon-experienced (n=39) | ND ^c^ | ND ^c^ | ND ^c^ |

^a^ Three patients (n=3) of genotype 3 with F0-2 had poor study protocol adherence, including premature withdrawal from treatment for unknown reasons (n=1), voluntary interruption of self-dosing between treatment weeks 2 and 4 (n=1), and loss-to-follow-up at post-treatment week 12 due to institutionalization (n=1); ^b^ 95% confidence interval not calculated for the proportion with a denominator below 10; ^c^ no stratified analysis planned for interferon-experienced patients by liver fibrosis. Data are in % (n/N) [95% confidence interval] using the Clopper-Pearson method. ND, not done.

**Supplementary Table S6.** Univariate and multivariate logistics regression analyses of genotype and fibrosis associated with SVR12 in FAS.

| **Variables** | **Univariate analysis** | | | **Multivariate analysis** | | |
| --- | --- | --- | --- | --- | --- | --- |
|  | **OR** | **95%CI** | **p-value^*^** | **OR** | **95%CI** | **p-value^*^** |
| Genotype |  |  |  |  |  |  |
| 1 | 1.00 (reference) |  |  | 1.00 (reference) |  |  |
| 2 | 3.92 | 0.70, 21.8 | 0.050 | 3.84 | 0.69, 21.4 | 0.067 |
| 3 | 9.89 | 1.86, 52.6 | **0.002** | 10.6 | 1.97, 56.9 | **<0.001** |
| 3a | 8.48 | 1.13, 63.4 | **0.002** | 9.01 | 1.20, 67.9 | **<0.001** |
| 3b | 11.1 | 1.77, 70.0 | **<0.001** | 12.0 | 1.88, 76.6 | **<0.001** |
| 6 | 1.98 | 0.18, 22.3 | 0.235 | 1.89 | 0.17, 21.4 | 0.265 |
| Fibrosis |  |  |  |  |  |  |
| F0-2 | 1.00 (reference) |  |  | 1.00 (reference) |  |  |
| F3 | 0.60 | 0.08, 4.88 | 0.296 | 0.46 | 0.06, 3.88 | 0.215 |
| F4 | 1.63 | 0.34, 7.85 | 0.424 | 1.61 | 0.32, 8.03 | 0.443 |

*Boostrapping approach. SVR12, sustained virologic response at post-treatment week 12; FAS, full analysis set; OR, odds ratio; CI, confidence interval.

**Supplementary Table S7.** Virologic failures and baseline/treatment-emergent NS5A/NS5B polymorphisms.

| **Type of virologic failure^*^** | **On-/post-treatment** | **GT** | **Fibrosis** | **RASs** | | | |
| --- | --- | --- | --- | --- | --- | --- | --- |
|  |  |  |  | **NS5A** | | **NS5B** | |
|  |  |  |  | **Baseline** | **On failure** | **Baseline** | **On failure** |
| Relapse | Post-treatment week 12 | 1a | F0-2 | ND | ND | ND | ND |
| Relapse ^a^ | Post-treatment week 4 | 1b | F0-2 | Q54H  Y93H | Assay failure | C316N  A338V | Assay failure |
| Relapse ^b^ | Post-treatment week 12 | 1b | F4 | Y93H | Assay failure | C316N  A338V | Assay failure |
| Relapse | Post-treatment week 4 | 2a | F0-2 | L31M | L31M | WT | WT |
| Relapse | Post-treatment week 4 | 2a | F0-2 | L31M | L31M | WT | WT |
| Relapse | Post-treatment week 4 | 2a | F0-2 | S25T  L31M  C92S | S25T  L31M  C92S | M289M/V | M289M/V |
| Relapse | Post-treatment week 4 | 2a | F0-2 | F28V  L31M | F28V  L31M | L392I | L392I |
| **Relapse ^c^** | **Post-treatment week 4** | **3a** | **F0-2** | **ND** | **ND** | **ND** | **ND** |
| Relapse | Post-treatment week 4 | 3b | F3 | ND | ND | ND | ND |
| Relapse | Post-treatment week 4 | 3b | F4 | ND | ND | ND | ND |
| Relapse | Post-treatment week 12 | 6n | F0-2 | ND | ND | ND | ND |
| Relapse | Post-treatment week 24 | 6e | F0-2 | ND | ND | ND | ND |
| Breakthrough ^d^ | On-treatment week 2 | 3b | F3 | ND | ND | ND | ND |
| **Lost-to-followup ^e^** | **Post-treatment week 12** | **3a** | **F0-2** | **ND** | **ND** | **ND** | **ND** |
| **Premature withdrawal ^f^** | **On-treatment week 2** | **3b** | **F0-2** | **ND** | **ND** | **ND** | **ND** |

^*^ All patients were interferon-naïve;

^a^ with confirmed hepatitis C virus ribonucleic acid (HCV RNA) below lower limit of quantitation at post-treatment week 12;

^b^ with confirmed occurrence of hepatocellular carcinoma at post-treatment week 12;

^c^ achieved virologic response at treatment week 2 →voluntary interruption of study drug dosing between treatment weeks 2 and 4 →with re-detected HCV RNA at treatment week 4 →continuation of study drug dosing →achieving virologic response at treatment weeks 8 and 12 →relapsed at post-treatment week 4;

^d^ achieved SVR12;

^e^ institutionalized but with sustained virologic response at post-treatment week 4;

^f^ for unknown reason but with virologic response at the last visit before lost-to-followup.

Bold fonts indicate poor on-treatment or post-treatment followup compliance.

GT, genotype; ND, not done; NS5A, nonstructural protein 5A; NS5B, nonstructural protein 5B; RASs, resistance-associated substitutions.

**Supplementary Table S8.** SVR12 with coblopasvir plus sofosbuvir in pooled phase 2/3 studies *versus* with velpatasvir-sofosbuvir in China phase 3 study (not head-to-head).

| **SVR12** | **Coblopasvir + sofosbuvir**  **(n=481) ^*^** | **Velpatasvir-sofosbuvir**  **(n=264)** ^†^ |
| --- | --- | --- |
| Overall | 467 (97%) | 254 (96%) |
| Genotype 1 | 247/249 (99%) | 82/82 (100%) |
| Genotype 2 | 117/122 (96%) | 61/61 (100%) |
| Genotype 3 | 52/57 (91%) | 49/59 (83%) |
| *Genotype 3a*  *Genotype 3b* | 24/26 (92%)  28/31 (90%) | 20/22 (91%)  29/37 (78%) |
| Genotype 6 | 51/53 (96%) | 62/62 (100%) |
| Compensated cirrhosis | 48/51 (94%) | 45/52 (87%) |
| Subset analysis |  |  |
| Overall (compliant) | 467/477 (98%) ^a^ | 254/263 (97%) ^b^ |
| Genotype 2 (compliant) | 117/121 (97%) | 61/61 (100%) |
| Genotype 3 (compliant) | 52/54 (96%) | 49/58 (84%) |
| *Genotype 3a (compliant)*  *Genotype 3b (compliant)* | 24/24 (100%)  28/30 (93%) | 20/21 (95%)  29/37 (78%) |
| Genotype 3 with cirrhosis | 5/6 (83%) | 10/17 (59%) |
| *Genotype 3a with cirrhosis*  *Genotype 3b with cirrhosis* | 3/3 (100%)  2/3 (67%) | 3/3 (100%)  7/14 (50%) |

^*^ China phase 2 study results also included: Rao H, et al. Safety and efficacy of coblopasvir and sofosbuvir in patients with genotypes 1, 2, 3 and 6 HCV infections without or with compensated cirrhosis. J Viral Hepat. 2020;27(1): 45-51.

^†^ China phase 3 study results included in Asian-Pacific multi-region phase 3 study: Wei L, et al. Sofosbuvir-velpatasvir for treatment of chronic hepatitis C virus infection in Asia: a single-arm, open-label, phase 3 trial. Lancet Gastroenterol Hepatol. 2019;4(2):127-134.

^a^ Four noncirrhotic patients (genotype 2a, n=1; 3a, n=2; 3b, n=1) with poor study compliance;

^b^ one noncirrhotic patient of genotype 3a with poor study compliance.

SVR12, sustained virologic response at post-treatment week 12.

**SUPPLEMENTARY FIGURES**

| 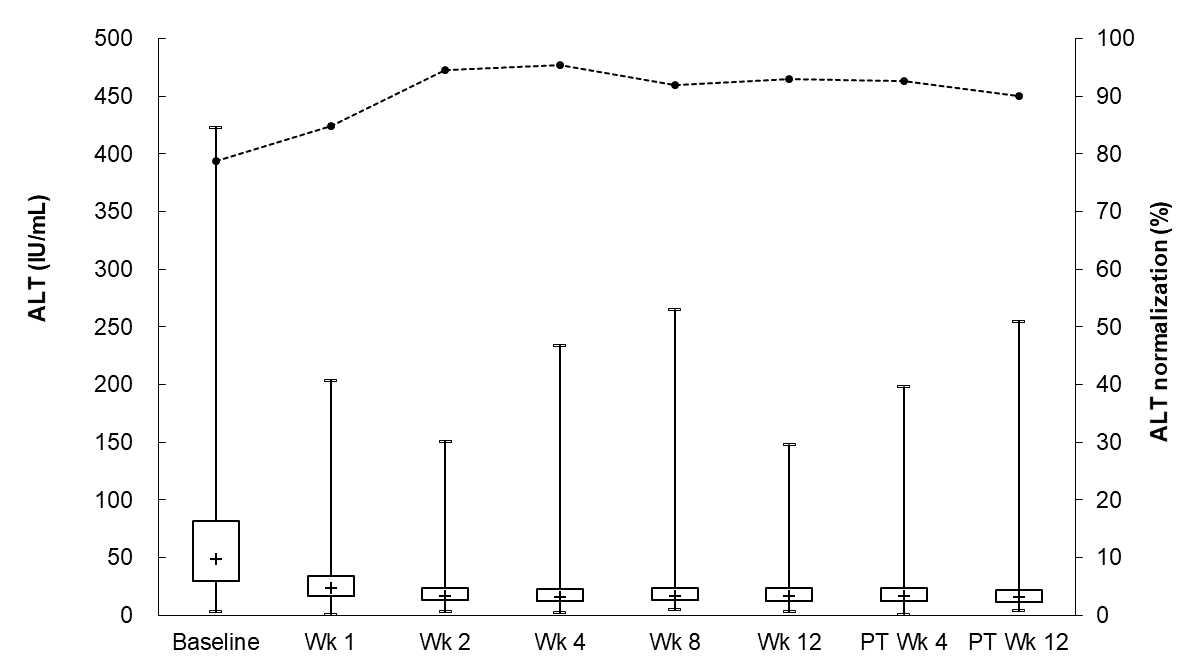 | 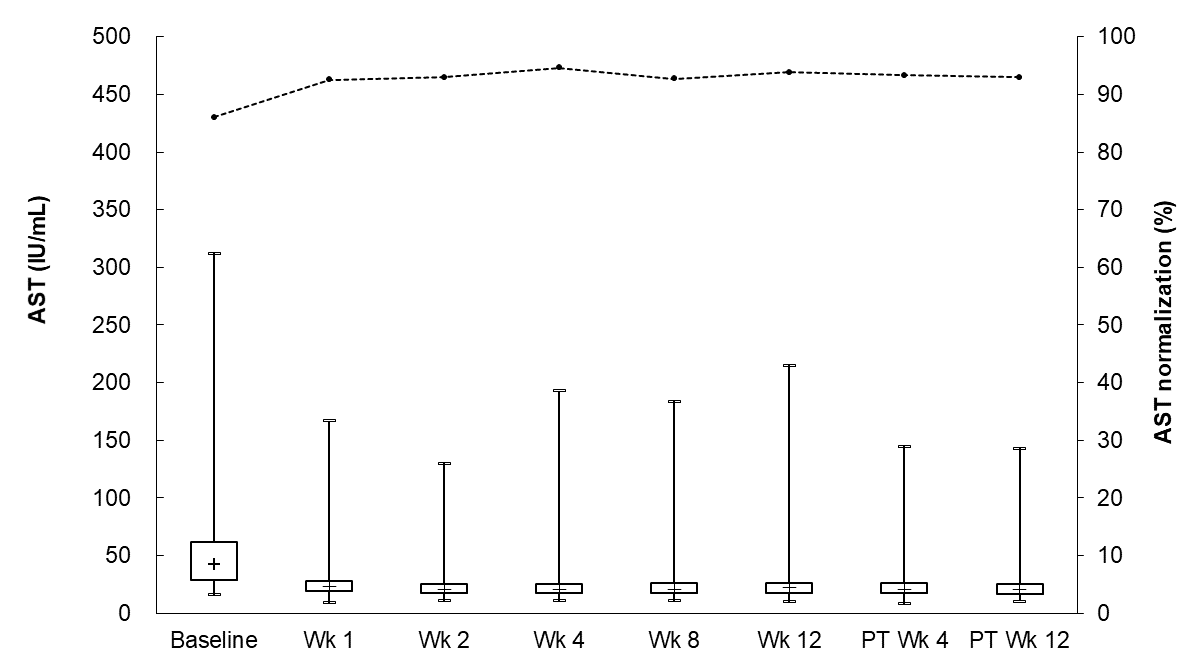 |
| --- | --- |
| 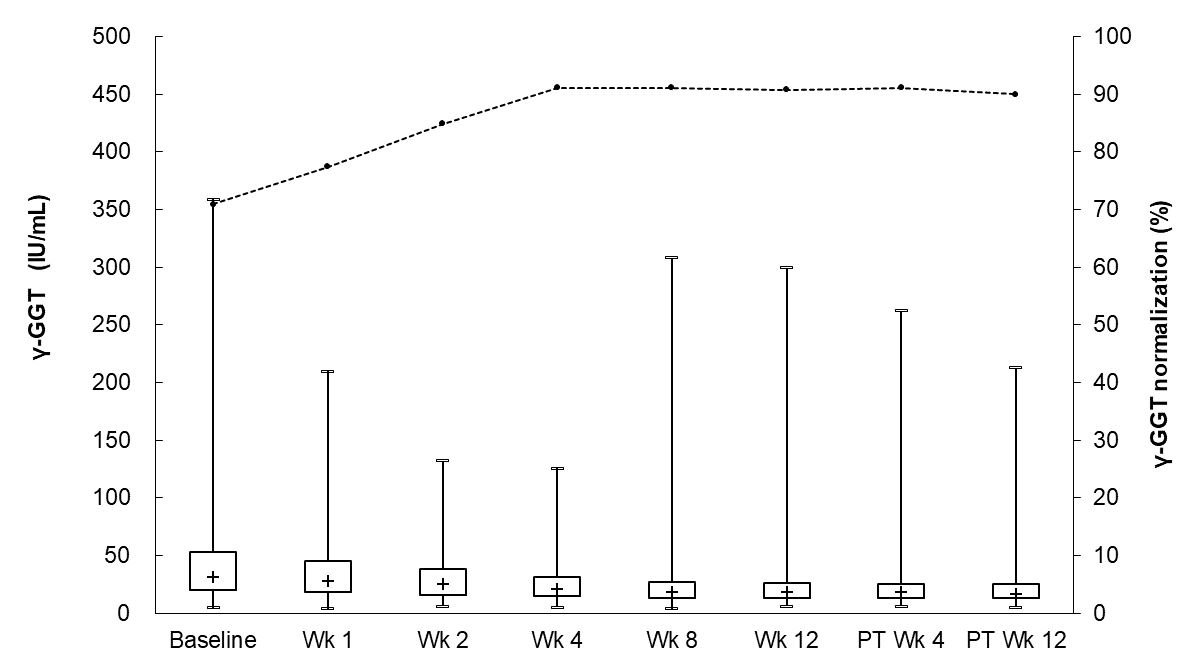 | 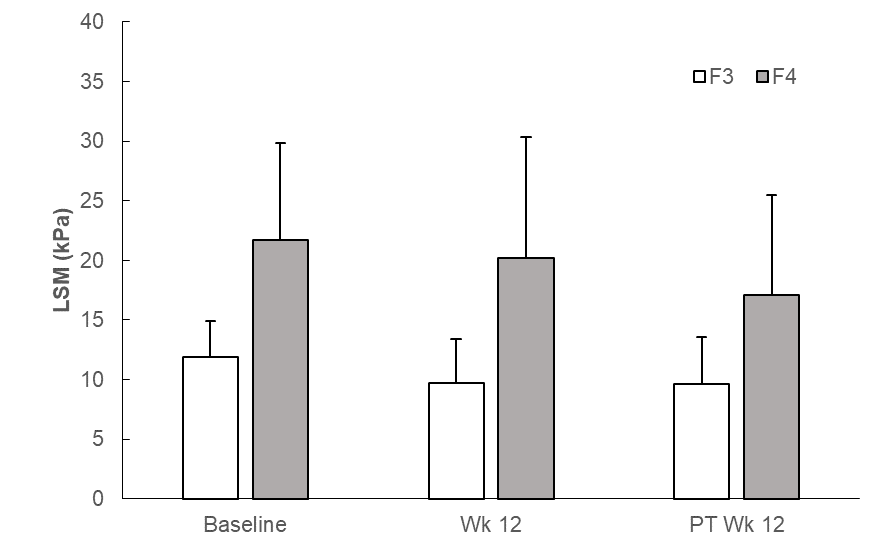 |

**Supplementary Figure 1.** Liver function tests and liver transient elastography. (A) Alanine aminotransferase (ALT); (B) aspartate aminotransferase (AST); (C) gamma-glutamyl transferase (γ-GGT); and (D) liver stiffness modulus (LSM). In the box-and-whisker plots (A-C), the box represents the interquartile range (from the 25^th^ to 75^th^ percentile) with the plus sign representing the median and the whiskers representing the maximum and the minimum, respectively; the dotted line represents the proportion of patients with a normalized liver function test. In the bar chart (D), the column represents the arithmetic mean with the bar representing the standard deviation. Baseline, on pre-treatment screening; Wk, week; PT, post-treatment.
